# Blood Pressure and Heart Rate Stationarity in Predicting TEVAR Response

**DOI:** 10.64898/2026.10.12.26365335

**Published:** 2026-10-13

**Authors:** Amira Alakhdar, Newell R. Washburn, Luka Pocivavsek, Nicole L. Bohr

## Abstract

**Background:**

Hypertension is a major risk factor for type B aortic dissections and rupture, with guidelines recommending systolic blood pressure (SBP) control below 130 mmHg to improve outcomes. While post-TEVAR blood pressure is often managed with a focus on mean SBP, little is known about temporal hemodynamic patterns and their association with surgical success. This study investigates blood pressure and heart rate (HR) time-series metrics within 24 hours post-TEVAR and their predictive potential for outcomes.

**Methods:**

Blood pressure (n = 560) and HR (n = 680) time-series data from 31 patients were analyzed alongside preoperative aortic geometry metrics. Exploratory data analysis identified correlations between BP variability and outcomes. Stationarity tests were performed on time-series data to assess statistical properties. Logistic regression models incorporating hemodynamic metrics, along with CT-derived features, were used to predict TEVAR outcomes.

**Results:**

Successful outcomes correlated with greater diastolic blood pressure variability and strictly stationary SBP. Logistic regression models combining these metrics along with heart rate standard deviation and preoperative aortic geometry achieved 96.67% 5-fold cross-validation accuracy in predicting outcomes.

**Conclusion:**

Post-TEVAR behaviors of hemodynamic variables are critical predictors of TEVAR success, with higher DBP standard deviation and strict stationarity of SBP significantly associated with favorable outcomes. These findings highlight the importance of hemodynamic variability and stationarity in optimizing post-TEVAR patient care.

